# Effect of internal connection parameters on the fatigue limit of narrow-diameter implants – a finite element study

**DOI:** 10.1016/j.jdent.2026.106510

**Published:** 2026-01-15

**Authors:** Renan Brandenburg dos Santos, Ulysses Lenz, Jason Alan Griggs, Alvaro Della Bona

**Affiliations:** a Postgraduate Program in Dentistry, School of Dentistry, University of Passo Fundo, Passo Fundo, RS, Brazil; b Department of Biomedical Materials Science, University of Mississippi Medical Center, MS, USA

**Keywords:** Dental implants, Finite element analysis, Fatigue

## Abstract

**Objective:**

To evaluate the influence of internal connection parameters on the fatigue limit of narrow-diameter dental implants using finite element analysis (FEA).

**Methods:**

A narrow-diameter dental implant (3.0 mm, Morse taper connection) and its corresponding abutment and screw were scanned using micro-computed tomography (micro-CT). High-resolution 3D models were reconstructed (Simpleware, Synopsis), and six geometric parameters defining the internal connection were extracted. A reference 3D assembly was built in SolidWorks, and systematic dimensional variations (±20%) of the six design parameters were introduced based on a Taguchi orthogonal array (DOE++, Reliasoft), resulting in 27 modified assemblies. Static FEA was performed in ABAQUS according to ISO 14,801:2016 geometry. Fatigue performance was estimated using Fe-safe software, and sensitivity analyses were conducted to identify the most influential parameters on the predicted fatigue limit.

**Results:**

The reference model presented a fatigue limit of 193 N. Dimensional optimization based on FEA improved the fatigue limit up to 295 N, representing a 52.3% increase compared to the original design, with the cervical opening diameter (CID) showing a significant effect on fatigue performance.

**Conclusions:**

Systematic variation of internal connection geometry significantly affected the fatigue resistance of narrow-diameter implants. Identifying critical parameters enables the development of more durable and reliable implant–abutment assemblies, maximizing the treatment success in anatomically compromised sites.

**Clinical significance:**

Optimizing the internal geometry of narrow-diameter implants can improve load distribution, minimize mechanical complications, and increase the longevity of implant-supported rehabilitations in areas with reduced bone volume.

## 1. Introduction

Narrow-diameter dental implants (NDIs) are widely indicated in cases with limited tridimensional bone availability or in anatomically constrained sites where bone augmentation is neither appropriate nor feasible. Clinical studies with follow-up periods up to 5 years showed that, when appropriately indicated, narrow-diameter implants are a reliable treatment option with satisfactory clinical outcomes [1–3]. This minimally invasive alternative reduces treatment time and avoids regenerative procedures while maintaining predictable outcomes. Nevertheless, when subjected to increased occlusal demands, particularly under off-axis loading conditions, NDIs may exhibit a reduced mechanical safety margin due to their smaller cross-sectional area. Therefore, the reduced cross-sectional area of NDIs imposes greater mechanical demands on the implant–abutment complex, increasing susceptibility to complications such as screw loosening, interfacial micromovement, and fatigue fracture under cyclic masticatory loads [4–7]. These risks are exacerbated under off-axis loading and marginal bone remodeling, which increase the lever arm of applied forces and concentrate stresses near the implant–abutment interface the most critical region for long-term mechanical stability [8,9].

The geometric configuration of the internal connection plays a key role in the biomechanical performance of dental implants by directly influencing stress dissipation and fatigue resistance. Various internal connection systems including conical, hexagonal, and hybrid designs exhibit distinct mechanical behaviors under cyclic loading, particularly in NDIs, where small geometric variations can significantly alter stress distribution [10,11]. Connections with higher taper angles tend to reduce micromovements and screw loosening, promoting improved long-term mechanical stability [12,13]. However, the geometric complexity of these interfaces also increases manufacturing challenges and makes failure prediction difficult, highlighting the need for accurate computational approaches to evaluate and optimize their structural behavior [14,15].

Finite Element Analysis (FEA) is widely recognized as a powerful tool in biomedical engineering for predicting the structural behavior of complex systems, enabling three-dimensional assessment of stresses, strains, and localized stress concentrations under controlled conditions [16]. This method provides a non-destructive and highly reproducible approach to study components that are difficult to evaluate experimentally, such as the implant–abutment interface, where failures typically initiate. In dentistry, FEA has been extensively used to analyze stress distribution across different implant macrogeometries, connection angles, and simulated bone conditions [11,17,18]. Furthermore, recent studies have demonstrated that small geometric modifications in critical regions—such as the internal cone and chamfer height—can substantially alter stress distribution and the predicted fatigue limit of the assembly [19,20]. The combined use of specialized software (ABAQUS, Fe-safe, SolidWorks, and DOE++) enables fatigue-life estimation in accordance with standardized protocols, such as ISO 14,801:2016 [21], providing results consistent with laboratory findings [22]. Therefore, FEA represents an essential tool for understanding and optimizing the mechanical behavior of narrow-diameter implants, supporting the development of new geometries with enhanced strength and clinical reliability. Therefore, the objective of this study is to evaluate the influence of internal connection geometry on the fatigue performance of narrow-diameter implants (NDIs) using a combined finite element analysis and fatigue simulation approach, testing the hypothesis that variations in specific geometric parameters of the internal connection affect stress distribution and, consequently, the fatigue resistance of NDIs.

## 2. Materials and method

### 2.1. Digitalization of structures

**Fig. 1.**
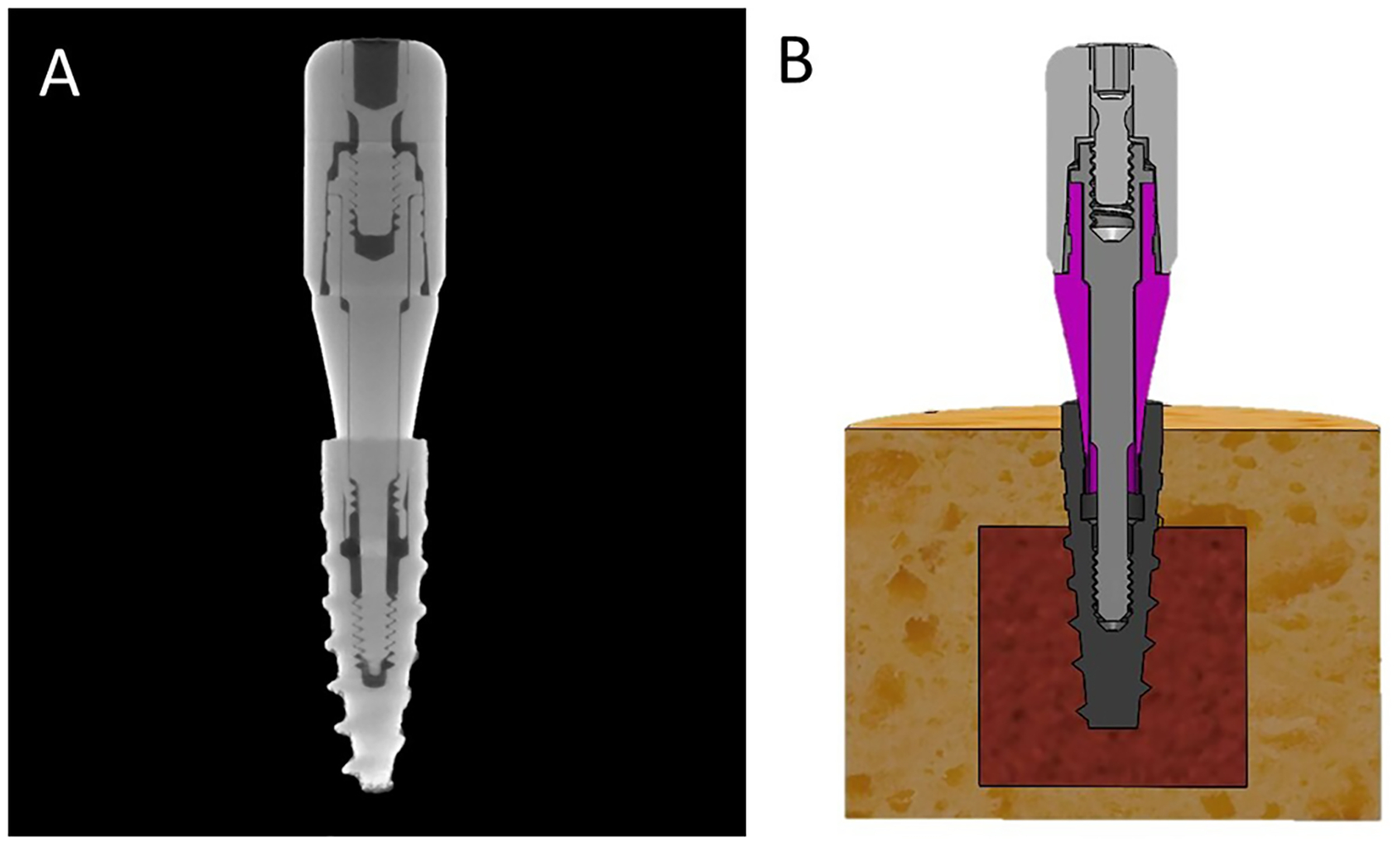
Bucco-palatal cross-sections illustrating the implant–abutment assembly. (A) Micro–computed tomography (micro-CT) image showing the physical implant–abutment complex. (B) Digital reconstruction of the same configuration modeled in SOLIDWORKS, including cortical and cancellous bone representation for finite element analysis.

An internal conical connection dental implant (Plenum^®^, Jundiaí, SP, Brazil), an abutment (Pilar Plenum, 2.5 mm transmucosal height), a connector screw, and a loading cap were scanned using micro-computed tomography (micro-CT) (Skyscan 1172; Micro Photonics, Allentown, PA, USA) with aluminum and copper filters and a pixel size of 34.6 μm. The resulting 3D bitmap files were processed with a grayscale thresholding tool in Simpleware W-2024.12 (Synopsis, Sunnyvale, CA, USA) to generate digital replicas of the physical specimens. The reconstructed 3D models, together with measurements obtained from an optical microscope VHX-1000 (Keyence Corporation, Osaka, Japan), were used to define the geometry of the components. A computer-aided design (CAD) software SOLIDWORKS (SOLIDWORKS 2023, D’assault Systèmes, Vélizy-Villacoublay, France) was then employed to digitally reproduce the specimens according to the measured dimensions using a reverse-engineering approach. A simulated specimen holder with cortical and cancellous bone layers was constructed [15,18,19,23,24]. The SOLIDWORKS components were assembled to replicate the physical configuration, with the implant positioned 0.8 mm above the cortical bone level to represent a worst-case scenario for internally tapered connections. This type of implant connection is generally recommended to be placed below the bone level, but subsequent bone resorption may occur. The final height of the implant was adopted considering a cohort study of 10, 871 implants with 8–10 years of follow-up that reported a mean bone loss of 0.49 ± 0.74 mm [25]. The final SOLIDWORKS assembly and a representative 2D image obtained from the micro-CT scan of the physical assembly are presented in Fig. 1.

### 2.2. Design of experiments

**Fig. 2.**
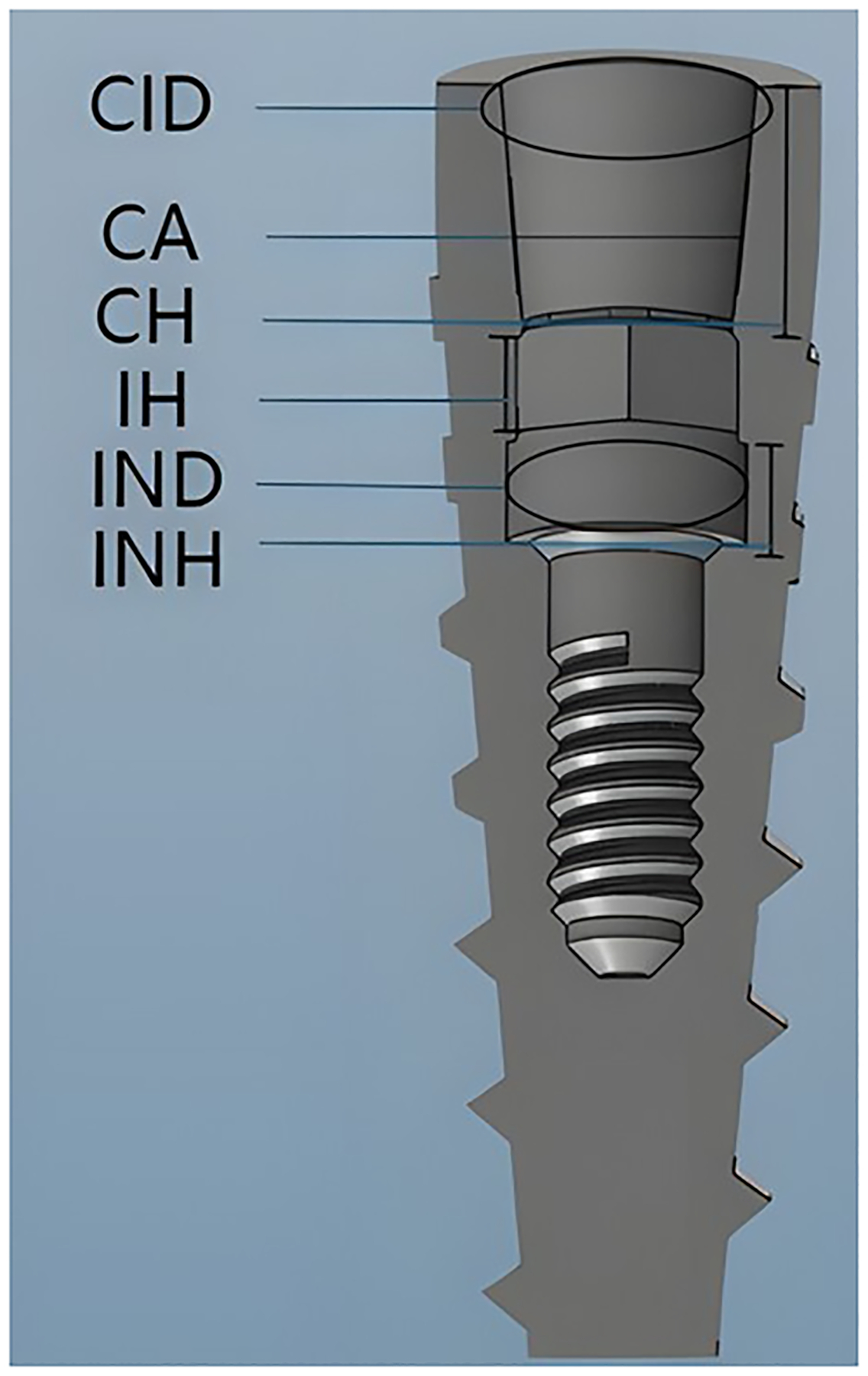
Schematic representation of the six internal connection parameters evaluated. CID (cervical inner diameter); CA (cone angulation); CH (cone height); IH (index height); IND (internal notch diameter); INH (internal notch height).

The internal geometry of the reference implant was characterized to identify critical design parameters affecting implant-abutment stability and fatigue performance. Six independent parameters were defined, each representing a distinct geometric feature of the internal connection (Fig. 2). These values were obtained from physical specimens (optical microscopy) and digital replicas (SOLIDWORKS) and set as baseline references. To investigate their effect, each parameter was systematically varied by ±20 %, establishing three levels per factor. However, the variation on the cervical opening diameter (CID) was limited to ±10 % to ensure clinical feasibility and to maintain the external diameter of the implant unchanged. The design parameters and their respective values are summarized in Table 1. A Design of Experiments (DOE) approach was performed in Reliasoft (DOE++ 2023, ReliaSoft Corporation, Tucson, AZ, USA) using a Taguchi orthogonal array, resulting in 27 unique abutment configurations. Each design was modeled in SOLIDWORKS and assembled with the reference components (simulated cortical and cancellous bone, implant body, connector screw, and loading cap) to evaluate fatigue performance. In addition, as the 27 models of the internal implant connection were systematically modified, the corresponding abutments were also adapted to reflect these connection pattern variations. Analysis of variance (ANOVA) was used to determine the parameters with significant influence on fatigue resistance (α = 0.05). Variance estimation followed Lenth’s method, in which variance is calculated as 1.5 times the median of all effects smaller than 2.5s_0_, with s_0_ defined as 1.5 times the median of all effects [26].

### 3.3. Virtual mechanical testing

Finite element analysis (FEA) was conducted in ABAQUS 2023 (Dassault Systèmes Simulia Corp., Johnston, RI, USA) in accordance with ISO 14801:2016. The SOLIDWORKS models were exported into ABAQUS, where material properties were defined as isotropic and homogeneous, with Young’s modulus and Poisson’s ratio assigned according to Table 2. Material property values were adopted from earlier experimental investigations reported in the literature [27–29]. A surface-to-surface interaction with a friction coefficient of 0.2 was applied between the transfixing screw and implant contact surfaces. The implant–abutment interface at the Morse taper connection was modeled as a frictional contact surface, but the preload applied to the transfixing screw is expected to minimize micromotion as in the clinical case of ‘cold welding’ [15,19,20,23]. Osseointegration was simulated by applying a tie constraint between implant threads and cortical and cancellous bone. Boundary conditions were imposed on the outer surface of the cortical bone to restrict movement under loading. An initial static load of 100 N was applied to the loading cap at a 30^◦^ angle relative to the implant axis. The assemblies were meshed using linear tetrahedral elements. A mesh convergence study was performed to balance accuracy and computational efficiency, resulting in an element size of 0.13 μm for critical components (implant, abutment, and connector screw). The reference model consisted of 125,110 elements.

The output database (.odb) files generated from FEA were imported into Fe-safe (Fe-safe 2023, Dassault Systèmes Simulia Corp., Johnston, RI, USA) for post-processing. Stress increments calculated in ABAQUS were used to simulate cyclic loading according to ISO 14801:2016, with a frequency of 2 Hz and a stress ratio of 0.1. Fatigue life of the implant–abutment assemblies was estimated using the Brown–Miller criterion with Morrow’s mean stress correction [30]. To establish the fatigue limit, cyclic loading began at 100 N, which resulted in infinite fatigue life. A subsequent 310 N load produced a finite fatigue life. Intermediate load levels within this range were then assessed to determine the threshold at which fatigue life transitioned from finite to infinite, thereby defining the maximum load under which the implant–abutment assembly would exhibit infinite fatigue life within the specified experimental conditions.

## 3. Results

**Fig. 3.**
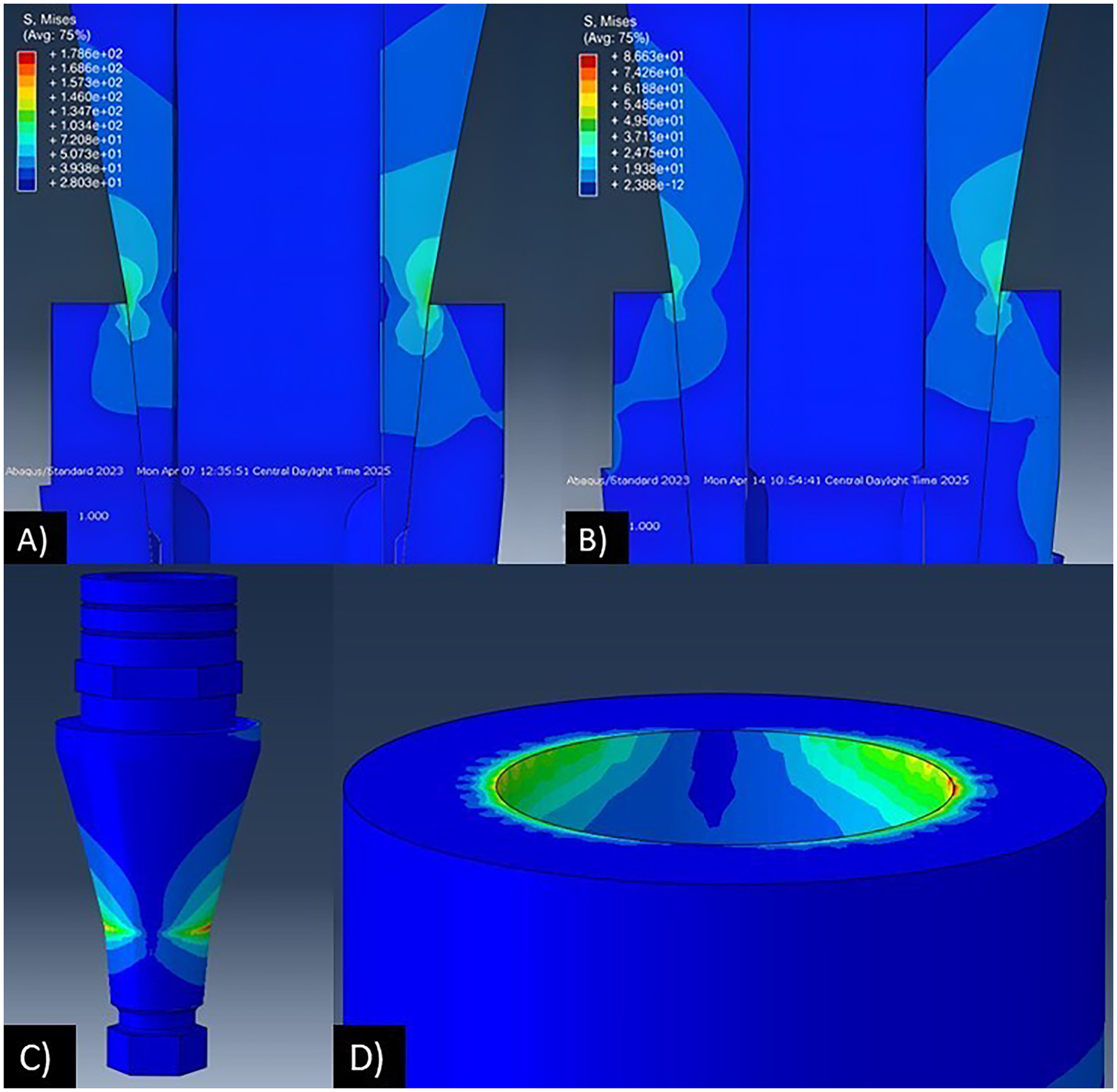
Finite element analysis (FEA) of von Mises stress distribution (in MPa) in implant–abutment assemblies. (A) Reference model showing a fatigue limit of 193 N. (B) Optimized configuration (Model 22) with a predicted fatigue limit of 295 N. (C) Abutment stress distribution (Model 4) showing peak stresses on the side opposite to the loading direction. (D) Implant stress distribution (Model 4) with similar stress localization on the opposite side of the applied load.

The mechanical performance of the 27 internal connection configurations was evaluated through finite element analysis (FEA) and fatigue simulations. The maximum von Mises stresses of the models subjected to a 100 N load were concentrated at the cervical region of the implant–abutment interface, mainly on the side opposite to the loading direction at the internal conical surface of the implant and the external conical surface of the abutment (Fig. 3). The peak stress values ranged from 82 MPa to 216 MPa among the evaluated configurations. The abutment, implant body, and connector screw, in this order, experienced the highest von Mises stress magnitudes within most assemblies. However, a shift in stress distribution was observed from Model 20 onward, in which both the implant and abutment shared the applied load more evenly, reducing localized stress peaks. This mechanical balance coincided with a reduction in maximum von Mises stress and a consistent increase in the predicted fatigue limit. The reference model presented a fatigue limit of 193 N, while the modified internal-connection designs showed limits ranging from 134 N to 295 N. Most models (59 %) exhibited higher fatigue limits than the reference, with the greatest improvement observed for Model 22, which reached a predicted fatigue limit of 295 N. The fatigue limits for the evaluated assemblies can be seen in Table 3.

**Fig. 4.**
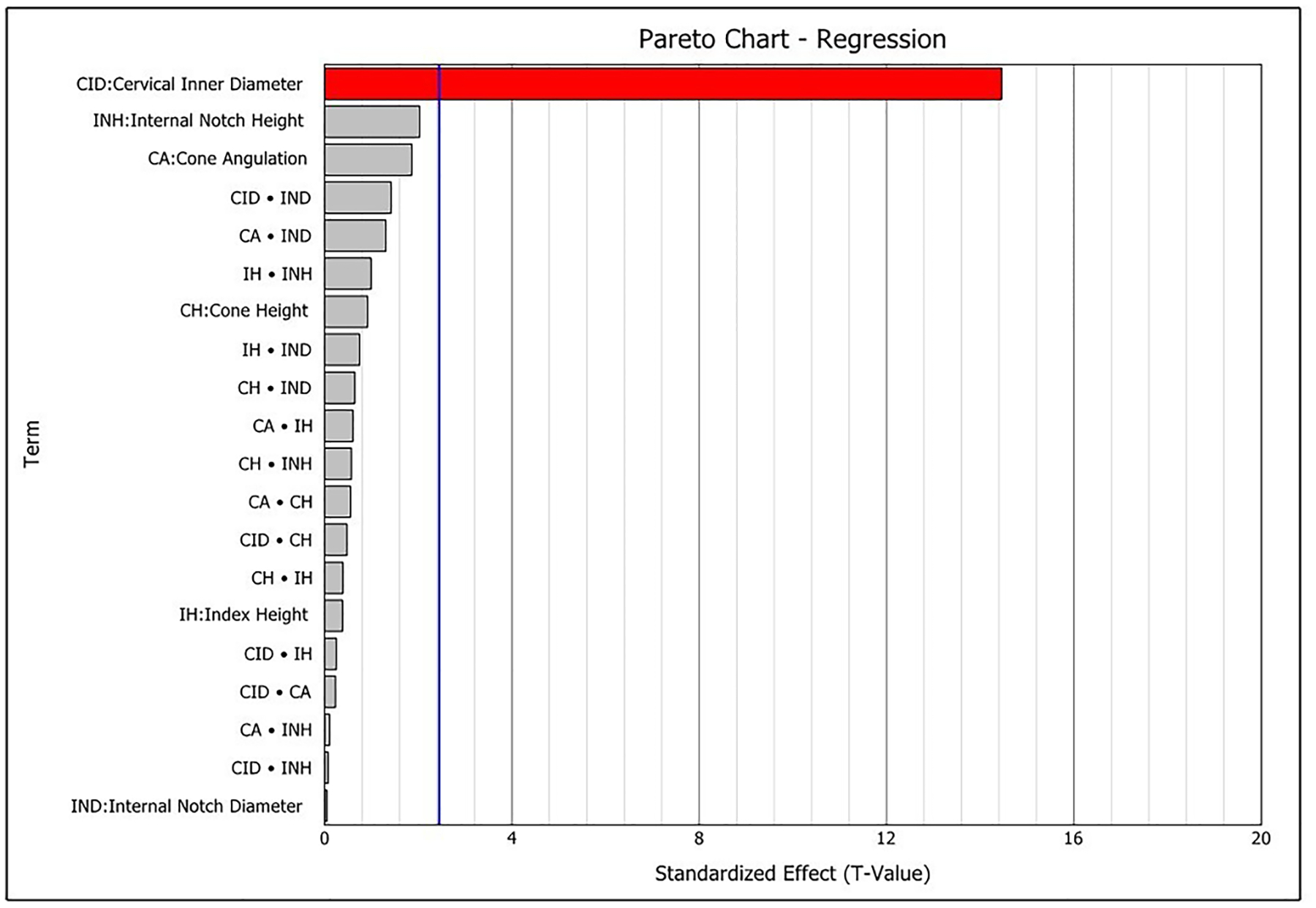
Pareto regression chart showing the standardized effects of the geometric parameters on the predicted fatigue limit of the implant–abutment assemblies. Bars extending beyond the significance threshold (T-value > 2.4) indicate parameters with a statistically significant influence (p ≤ 0.1). Abbreviations: CID- cervical inner diameter; CA- cone angulation; CH- cone height; IH- index height; IND- internal notch diameter; INH- internal notch height.

**Fig. 5.**
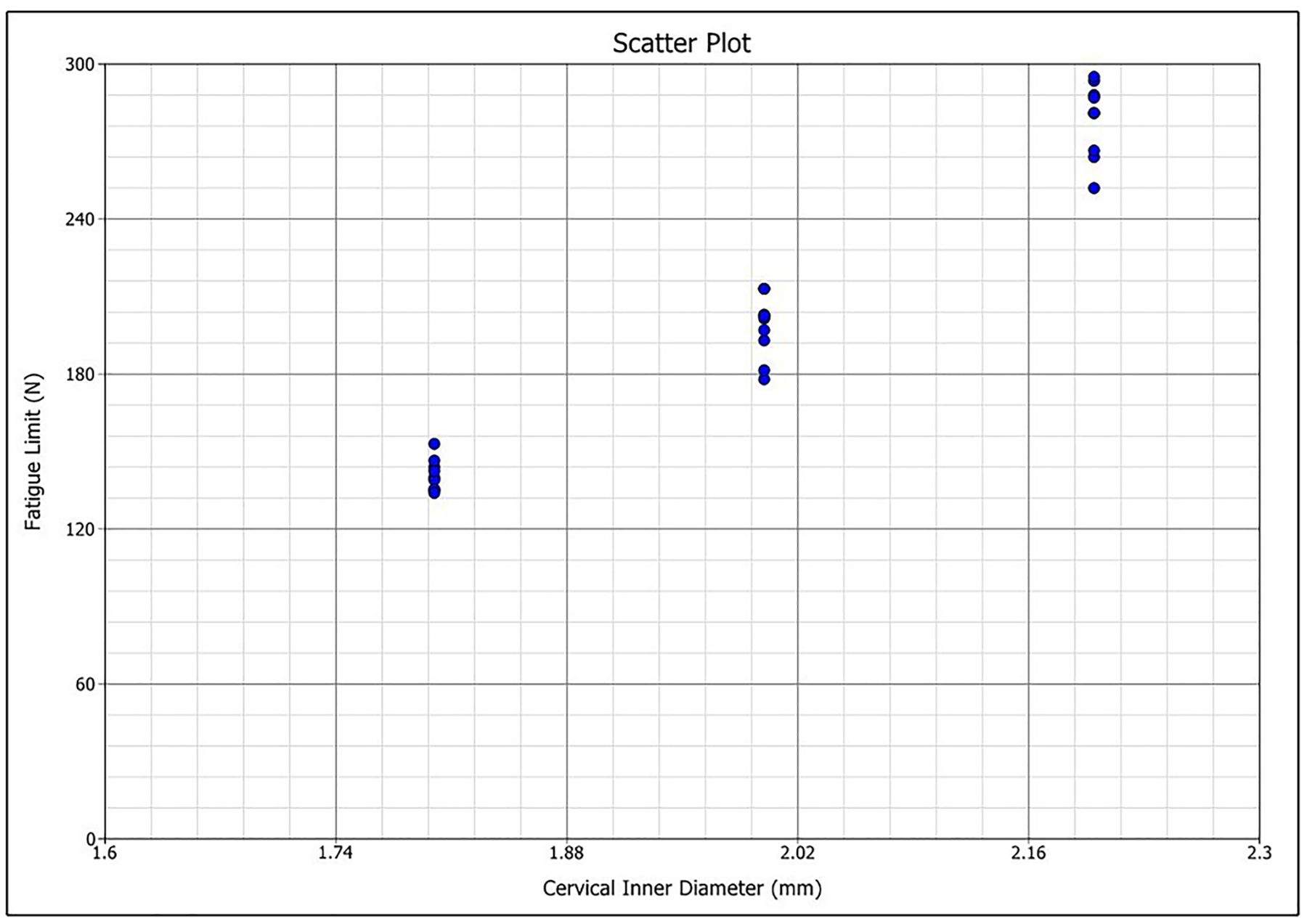
Scatter plot showing the relationship between the cervical inner diameter (CID) parameter and the predicted fatigue limit of the implant–abutment assemblies.

Analysis of variance (ANOVA) and multiple linear regression were performed to identify the geometric parameters that most influenced the fatigue behavior of the implant–abutment assemblies. Both analyses confirmed that the cervical opening diameter (CID) was the most significant factor affecting the fatigue limit, showing a highly significant effect (*p* ≤ 0.001) (Fig. 4). The index notch height (INH) exhibited a trend toward statistical significance (*p* = 0.09), suggesting that variations in this parameter may also affect stress distribution in the internal connection. The remaining factors cone angulation (CA), cone height (CH), index height (IH), and internal notch diameter (IND) did not show significant effects when considered singly (*p* > 0.1), but their combined effects caused approximately 20 % increase in fatigue limit (Fig. 5).

## 4. Discussion

The present study evaluated the influence of internal connection geometry on the fatigue performance of narrow-diameter implants (NDIs) using a combined finite element analysis and fatigue simulation approach. Among the six geometric parameters analyzed, the cervical opening diameter (CID) showed the strongest effect on the fatigue limit, as confirmed by ANOVA and multiple regression (*p* ≤ 0.001), partially accepting the study hypothesis. This finding highlights the critical role of internal geometry in determining the mechanical reliability of implant–abutment assemblies, especially in narrow-diameter implants where stress concentrations are more pronounced [6,15,31]. Previous studies have also demonstrated that small changes in the internal conical configuration can substantially modify stress distribution and fatigue life [32–34]. The results from the present work reinforce this evidence, showing that geometric optimization of the internal connection can significantly enhance the fatigue resistance of NDIs, potentially extending their clinical longevity.

Considering that the implant–abutment contact region is the area of highest masticatory load incidence, the structural behavior of this interface is critical for the mechanical longevity of the assembly [10,35]. The trend toward improved fatigue resistance observed with increasing cervical opening diameter (CID) may be associated with fundamental bioengineering principles, such as more efficient stress sharing and enhanced local structural stiffness. A larger internal diameter increases the load-bearing cross-section and the conical engagement area, favoring a more uniform stress dissipation and reducing critical stress concentrations at the interface [36,37]. This geometric modification also tends to promote a smoother transition of forces along the conical walls, mitigating shear stresses under eccentric loading conditions. From a biomechanical standpoint, enlarging the supporting region and increasing the volume of resistant material may delay the onset of localized plasticity and crack initiation under cyclic loading, a finding consistent with previous mechanical and dental engineering studies [11,30,38].

A relevant biomechanical improvement is observed in the models 19–23 (Fig. 5), which is aligned with the increasing in the cervical opening diameter (CID), resulting in a more homogeneous stress distribution within the implant–abutment assembly. This redistribution of mechanical demands reduced the magnitude of critical von Mises stresses and prevented localized overloads, particularly at the abutment neck and implant platform. Such behavior indicates improved load transfer efficiency across the connection, promoting mechanical equilibrium and enhancing the overall fatigue limit of the system. Similar phenomena have been reported in studies on internal conical connections, where a more homogeneous stress distribution strongly correlates with increased fatigue resistance and longer predicted lifespans [6,19,39]. Although other parameters such as index notch height (INH) and cone height (CH) did not reach statistical significance, their subtle variations may complement the mechanical optimization observed, contributing to localized adjustments in stress flow within the assembly, as confirmed by previous studies [40,41].

Recent advances in implant design have demonstrated a growing industrial trend toward the use of wider internal taper angles, which, when combined with an increased cervical opening diameter (CID), can enhance the mechanical efficiency of the implant–abutment interface. Larger taper angulations promote a more uniform dissipation of stresses and facilitate a more precise and stable adaptation between components, thereby reducing the occurrence of microgaps and micromovements during functional loading [42,43]. This geometric configuration also increases the material volume in the abutment’s cervical region, reinforcing the most vulnerable portion of the connection and minimizing the risk of mechanical failure under cyclic loading [44,45]. Therefore, the combination of wider taper angles and larger CID values represents a synergistic strategy for optimizing the mechanical strength of narrow-diameter systems, consistent with current industrial manufacturing trends and bioengineering approaches aimed at improving internal connection performance [19,46].

The improvement in fatigue performance associated with increasing CID demonstrates that geometric refinements in the cervical region can optimize stress dissipation and enhance load transfer efficiency, even under critical loading conditions [47,48]. This finding supports the rationale that internal macrogeometric optimization can compensate for dimensional constraints imposed by reduced implant diameters, resulting in assemblies that are more stable and less prone to fatigue-related failure [15,23]. Therefore, structural optimization of the internal connection should not be regarded merely as a reinforcement strategy for NDIs but as a broader engineering concept applicable to the design of implants with enhanced clinical longevity and biomechanical predictability, regardless of their dimensions. This trend is consistent with recent studies demonstrating a direct relationship between internal geometric refinement, reduction of stress concentrations, and improved mechanical durability [5,7].

Although the present study was based on *in vitro* simulations, it provides a robust and controlled analysis of how internal connection geometry influences the mechanical performance of narrow-diameter implants. Finite element modeling allowed the isolation of individual geometric parameters under standardized conditions, ensuring consistency and reliability of the numerical data. Nevertheless, these results should be interpreted within the limitations of an *in vitro* investigation, which does not fully reproduce the complex biological, material, and loading conditions of the oral environment. In addition, the numerical analyses were restricted to a single implant design and a single abutment configuration; therefore, the external validity of the findings may be limited and the observed trends should be interpreted primarily within the investigated implant–abutment system. Consequently, extrapolation to other commercial implant designs, connection concepts, and prosthetic configurations should be made with caution. Future experimental and clinical studies are, therefore, essential to validate these findings, enabling direct comparisons with real prosthetic restorations and long-term clinical performance. Collaboration between academic research and the implant industry will be crucial to translate these mechanical insights into optimized implant designs with enhanced fatigue resistance, reliability, and clinical predictability.

## 5. Conclusion

Considering the results of the study, it can be concluded that:

- Increasing the cervical opening diameter (CID) significantly enhanced the fatigue resistance of narrow-diameter implant/abutment assemblies, highlighting the direct impact of internal connection geometry on mechanical performance.
- The reduction in maximum von Mises stresses observed in the optimized models indicated a more balanced force distribution along the implant–abutment interface, contributing to higher fatigue limits and improved structural stability of the system.

## Figures and Tables

**Table 1:** Design parameters and their respective values.

Design	Parameters	20 % Lower	Reference	20 % Higher
CID	Cervical Inner Diameter	1.8 mm*	2.0 mm	2.2 mm*
CA	Cone Angulation	8.8°	11.0°	13.2°
CH	Cone Height	1.6 mm	2.0 mm	2.4 mm
IH	Index Height	0.64 mm	0.80 mm	0.96 mm
IND	Internal Notch Diameter	1.50 mm	1.88 mm	2.25 mm
INH	Internal Notch Height	0.48 mm	0.60 mm	0.72 mm

* The cervical opening diameter (CID) was limited to ±10 % variation to preserve the main implant diameter and to ensure clinical feasibility.

**Table 2:** Material properties assigned to each component used in the finite element models.

Component	Material	Young’s Modulus (GPa)	Poisson’s Ratio	Ultimate Tensile Strength (MPa)
Implant Body	Ti6A4V-ELI	113.8	0.31	825
Abutment	CPTiGrade4	103	0.34	660
Connector Screw	Ti6A4V-ELI	113.8	0.31	825
Cap	Ti6AlV-ELI	105	0.31	825
Cancellous Bone	Cancellous Bone	14	0.3	-
Cortical Bone	Cortical Bone	20	0.3	-

Ti: Titanium; Al: Aluminum; V: Vanadium; ELI: extra low interstitial; CP: commercially pure.

**Table 3:** Maximum von Mises stress values (MPa) under a 100 N load and predicted fatigue limits (N) for the evaluated assemblies, along with their respective design parameters (mm).

Evaluated models	Cervical open diameter (CID)	Cone Angulation (CA)	Cone Height (CH)	Index Height (IH)	Internal Notch Diameter (IND)	Internal Notch Height (INH)	Peak von Mises stress	Fatigue limit
REFERENCE	2.0	11.0°	2.0	0.80	1.88	0.60	178	193
1	1.8	13.2°	2.4	0.80	2.25	0.48	183	142.5
2	1.8	13.2°	1.6	0.96	2.25	0.60	148	134
3	1.8	11.0°	2.4	0.64	1.88	0.60	215	146.5
4	1.8	8.8°	2.0	0.80	1.50	0.60	153	153
5	1.8	11.0°	1.6	0.80	1.88	0.72	149	135.5
6	1.8	11.0°	2.0	0.96	1.88	0.48	211	135
7	1.8	8.8°	1.6	0.64	1.50	0.48	155	139
8	1.8	13.2°	2.0	0.64	2.25	0.72	220	140
9	1.8	8.8°	2.4	0.96	1.50	0.72	167	144
10	2.0	11.0°	2.0	0.64	1.50	0.72	118	178
11	2.0	8.8°	2.0	0.96	2.25	0.48	158	202.5
12	2.0	13.2°	2.0	0.80	1.88	0.60	124	181,5
13	2.0	8.8°	1.6	0.80	2.25	0.72	152	193
14	2.0	11.0°	2.4	0.80	1.50	0.48	101	213
15	2.0	13.2°	2.4	0.96	1.88	0.72	152	201.5
16	2.0	13.2°	1.6	0.64	1.88	0.48	113	213
17	2.0	11.0°	1.6	0.96	1.50	0.60	108	197
18	2.0	8.8°	2.4	0.64	2.25	0.60	112	203
19	2.2	11.0°	1.6	0.64	2.25	0.48	111	287
20	2.2	13.2°	2.0	0.96	1.50	0.48	99	281
21	2.2	8.8°	1.6	0.96	1.88	0.60	84	293.5
22	2.2	8.8°	2.4	0.80	1.88	0.48	83	295
23	2.2	11.0°	2.0	0.80	2.25	0.60	88	266.,5
24	2.2	8.8°	2.0	0.64	1.88	0.72	85	281
25	2.2	13.2°	1.6	0.80	1.50	0.72	82	252
26	2.2	11.0°	2.4	0.96	2.25	0.72	82	264
27	2.2	13.2°	2.4	0.64	1.50	0.60	86	288
